# Protein Signature Differentiating Neutrophils and Myeloid-Derived Suppressor Cells Determined Using a Human Isogenic Cell Line Model and Protein Profiling

**DOI:** 10.3390/cells13100795

**Published:** 2024-05-07

**Authors:** Yuting Zhang, Jin Hu, Xiashiyao Zhang, Minzhi Liang, Xuechun Wang, Dailin Gan, Jun Li, Xuemin Lu, Jun Wan, Shan Feng, Xin Lu

**Affiliations:** 1 Department of Biological Sciences, Boler-Parseghian Center for Rare and Neglected Diseases, Harper Cancer Research Institute, University of Notre Dame, Notre Dame, IN 46556, USA; yzhang49@nd.edu (Y.Z.); mliang3@nd.edu (M.L.); xwang53@nd.edu (X.W.); xlu1@nd.edu (X.L.); 2 Integrated Biomedical Sciences Graduate Program, University of Notre Dame, Notre Dame, IN 46556, USA; 3 Mass Spectrometry & Metabolomics Core Facility, Key Laboratory of Structural Biology of Zhejiang Province, Westlake University, Hangzhou 310024, China; hujing@westlake.edu.cn (J.H.); fengshan@westlake.edu.cn (S.F.); 4 Department of BioHealth Informatics, Luddy School of Informatics, Computing, and Engineering, Indiana University Indianapolis, Indianapolis, IN 46202, USA; xz108@iu.edu (X.Z.); junwan@iu.edu (J.W.); 5 Department of Applied and Computational Mathematics and Statistics, University of Notre Dame, Notre Dame, IN 46556, USA; dgan@nd.edu (D.G.); jun.li@nd.edu (J.L.); 6 Department of Medical and Molecular Genetics, Indiana University School of Medicine, Indianapolis, IN 46202, USA; 7 Center for Computational Biology and Bioinformatics, Indiana University School of Medicine, Indianapolis, IN 46202, USA; 8 Tumor Microenvironment and Metastasis Program, Indiana University Melvin and Bren Simon Comprehensive Cancer Center, Indianapolis, IN 46556, USA

**Keywords:** myeloid-derived suppressor cell, immunotherapy, mass spectrometry, proteomics, protein signature

## Abstract

Myeloid-derived suppressor cells (MDSCs) play an essential role in suppressing the antitumor activity of T lymphocytes in solid tumors, thus representing an attractive therapeutic target to enhance the efficacy of immunotherapy. However, the differences in protein expression between MDSCs and their physiological counterparts, particularly polymorphonuclear neutrophils (PMNs), remain inadequately characterized, making the specific identification and targeting of MDSCs difficult. PMNs and PMN-MDSCs share markers such as CD11b+CD14−CD15+/CD66b+, and some MDSC-enriched markers are emerging, such as LOX-1 and CD84. More proteomics studies are needed to identify the signature and markers for MDSCs. Recently, we reported the induced differentiation of isogenic PMNs or MDSCs (referred to as iPMNs and iMDSCs, respectively) from the human promyelocytic cell line HL60. Here, we profiled the global proteomics and membrane proteomics of these cells with quantitative mass spectrometry, which identified a 41-protein signature (“cluster 6”) that was upregulated in iMDSCs compared with HL60 and iPMN. We further integrated our cell line-based proteomics data with a published proteomics dataset of normal human primary monocytes and monocyte-derived MDSCs induced by cancer-associated fibroblasts. The analysis identified a 38-protein signature that exhibits an upregulated expression pattern in MDSCs compared with normal monocytes or PMNs. These signatures may provide a hypothesis-generating platform to identify protein biomarkers that phenotypically distinguish MDSCs from their healthy counterparts, as well as potential therapeutic targets that impair MDSCs without harming normal myeloid cells.

## 1. Introduction

Immunotherapy has emerged as a transformative approach in the battle against cancer, offering hope where traditional treatments have faltered [1]. However, its efficacy is not universal. Certain cancers, such as prostate cancer, exhibit notable de novo resistance to immunotherapy [2]. Myeloid-derived suppressor cells (MDSCs) have been identified as a critical contributor to immunosuppression in the tumor microenvironment [3,4]. Extensive studies have revealed MDSCs to be a heterogeneous population of cells that expand during cancer and other diseases and are characterized by their ability to suppress T cell responses and promote tumor growth [5]. The mechanisms by which MDSCs operate are diverse, encompassing the depletion of essential amino acids critical for T cell function, the production of reactive oxygen and nitrogen species, and the induction of regulatory T cells. Additionally, MDSCs interact with and influence other immune cells, contributing to a systemic alteration of the immune response in cancer patients [4,6].

Current strategies for targeting MDSCs in preclinical studies include depletion of existing MDSCs, blockade of the development of MDSCs, blockade of MDSC recruitment, or inhibition of MDSCs’ immunosuppressive potentials [7]. The common depletion approaches involve antibodies targeting known MDSC markers to induce NK cell-mediated antibody-dependent cellular cytotoxicity, such as the anti-Gr1 antibody [8,9,10] in mice or the anti-CD33 antibody [11,12] in humans. Unfortunately, these markers are also expressed by normal immune cells like neutrophils or monocytes, posing a risk of severe side effects such as neutropenia [13]. This overlap underscores the necessity for more specific MDSC markers that can be targeted without compromising the integrity of the immune system.

Considering these challenges, numerous studies have delved deeper into the phenotypic and functional traits that differentiate human or mouse MDSCs from their normal myeloid counterparts [6]. For instance, Bergenfelz et al. highlighted the unique expression profiles of monocytic MDSCs (Mo-MDSCs) in the milieu of human breast cancer, contrasting them with healthy monocytes [14]. Condamine et al. examined the distinctions between low-density polymorphonuclear MDSCs (PMN-MDSCs) and high-density neutrophils from head and neck cancer and lung cancer patients, identifying Lectin-type oxidized LDL receptor-1(LOX-1) as a discriminative marker for PMN-MDSCs [8]. These studies involving human cancers typically have two limitations: first, the MDSCs and neutrophils were isolated from different individuals, causing inherent variability; second, it is often unclear whether the differences detected were specific to the cancer type investigated or generally true. A number of proteomic studies on neutrophils or MDSCs are limited to murine samples [9,10] or to human neutrophils in non-cancer contexts [11,12,15]. These studies have provided excellent discovery methodologies and datasets for cross validations.

We took an approach that leveraged our previously established human isogenic cell line models of MDSCs and neutrophils using the human promyelocyte HL60 cell line [16]. Originating from an acute myeloid leukemia patient, HL60 serves as a classic in vitro model for human neutrophil induction [16,17]. Dimethyl sulfoxide (DMSO) is a standard differentiation reagent to induce neutrophil differentiation through several mechanisms, including the induction of cell cycle arrest at the G1/G0 phase, thus triggering differentiation [17,18,19,20]. By manipulating the presence or absence of the cytokine cocktail Granulocyte-macrophage colony-stimulating factor (GM-CSF) and interleukin 6 (IL-6), we successfully generated isogenic neutrophils and MDSCs [21]. GM-CSF plays a critical role in the proliferation and differentiation blockade of MDSCs [22,23,24], while IL-6 is instrumental in MDSC activation [25,26].

The purpose of this study is to identify protein signatures between MDSCs and their normal counterparts especially neutrophils, as potential diagnostic and prognostic tools. Certain specific proteins in the signature may be explored in the future as potential MDSC markers for depletion or other therapeutic opportunities. Our study performed proteomic profiling of the HL60-derived iPMNs and iMDSCs and compared the data with published data to yield potential MDSC-associated markers. A 38-protein signature has been identified that potentially enables a better distinction between MDSCs and their physiological counterparts.

## 2. Materials and Methods

### 2.1. Cell Culture

HL60 cells (ATCC, CCL-240, Manassas, VA, USA) were cultured in Iscove’s Modified Dulbecco’s Medium (IMDM, HyClone, GE Healthcare Life Sciences, SH30228.01, Marlborough, MA, USA) supplemented with 20% fetal bovine serum (FBS, HyClone, GE Healthcare Life Sciences SH30396.03, Marlborough, MA, USA) and 1X Penicillin/Streptomycin (P/S, Caisson Labs, PSL01, Smithfield, UT, USA), as recommended on ATCC website. Jurkat cells (clone E6-1, ATCC, TIB-152, Manassas, VA, USA) were initially cultured in RPMI 1640 with 10% FBS and 1X P/S and gradually changed into IMDM supplemented with 10% FBS and 1X P/S. The cells were all cultured between 1 × 10^5^ cells/mL and 1 × 10^6^ cells/mL. Cell counting was conducted by Invitrogen Countess 3 Automated Cell Counter (Fisher Scientific, A49865, Waltham, MA, USA). Cell viabilities were assessed by Trypan Blue solution (VWR, 45000-717, Radnor, PA, USA). All cells were tested to confirm that they were free of mycoplasma by the Mycoplasma Assay Kit (Agilent Technologies, 302109, Santa Clara, CA, USA) every two weeks.

### 2.2. iPMN and iMDSC In Vitro Induction

The generation of induced polymorphonuclear cells (iPMN) was achieved through the treatment of HL60 cells with 1.25% dimethyl sulfoxide (DMSO) (Sigma-Aldrich, D4540, St. Louis, MO, USA) over a period of six days (day 0 to day 6). The induction of myeloid-derived suppressor cells (iMDSC) commenced with 1.25% DMSO for the initial six days (day −6 to day 0), followed by the supplementation of the culture medium with 10 ng/mL GM-CSF (Tonbo Biosciences, 21-8339-U020, San Diego, CA, USA) and 10 ng/mL IL-6 (Tonbo Biosciences, 21-8069-U020, San Diego, CA, USA) for an additional six days (day 0 to day 6). To ensure optimal conditions, the medium was replenished with fresh culture medium containing GM-CSF and IL-6 on day 3. Throughout the culturing process, the density of both iPMN and iMDSC populations was meticulously regulated to remain within the range of 1 × 10^5^ to 1 × 10^6^ cells/mL.

### 2.3. Jurkat Interleukin-2 (IL-2) Measurement to Evaluate Immunosuppression

The experimental setups for stimulating Jurkat cells, as well as their co-culture with HL60, iPMN, or iMDSC, followed established protocols [21]. Jurkat cells were activated using the Immunocult Human CD3/CD28 T Cell Activator (STEMCELL Technologies, 10971, Cambridge, MA, USA). HL60, iPMN, or iMDSC were mixed with the Jurkat cells in a 1:1 ratio and incubated for 24 h in 96-well flat-bottom culture plates in IMDM supplemented with 10% FBS. Following incubation, cells were collected, centrifuged at 450× *g* for 10 min at 4 °C, and the culture supernatant was preserved at −80 °C for subsequent analysis. The concentration of IL-2 secreted was measured using a Human IL-2 ELISA kit (Biolegend, 431804, San Diego, CA, USA) according to the manufacturer’s instructions. To ensure reproducibility, the assay was conducted three times, with consistent results, and representative results were plotted.

### 2.4. Global Proteomics

Three biological replicates of each cell type were performed. Protein concentration in each sample was determined using the bicinchoninic acid (BCA) assay, setting aside 100 μg for downstream analysis. The proteins were first reduced with 10 mM dithiothreitol (DTT) at 50 °C for 45 min, followed by alkylation with 25 mM iodoacetamide (IAM) in the dark at ambient temperature for 30 min. Cold acetone was used for protein precipitation, after which the pellet was resolubilized in 100 mM triethylammonium bicarbonate (TEAB) and digested overnight with trypsin at 37 °C. The resulting peptides were labeled using TMT6 reagent, and the labeling was quenched with hydroxylamine. The TMT-labeled peptides from different samples were then pooled, cleaned with an HLB column (Waters), and separated into 10 fractions by high-pH reverse-phase chromatography [27].

For LC-MS/MS analysis, the peptides were eluted over a 120-min gradient at a flow rate of 0.300 μL/min on a Thermo UltiMate 3000 integrated nano-HPLC system interfaced with a Thermo Orbitrap Fusion Lumos mass spectrometer. Separation was achieved using a homemade fused silica capillary column (75 μm ID, 150 mm length; Upchurch, Oak Harbor, WA, USA) packed with C-18 resin (300 A, 3 μm, Varian, Lexington, MA, USA). The mobile phases consisted of 0.1% formic acid in water (phase A) and 80% acetonitrile with 0.1% formic acid (phase B). The mass spectrometer was operated in data-dependent acquisition mode, beginning with a full-scan mass spectrum in the Orbitrap (350–1800 *m*/*z*, 60,000 resolution) followed by MS/MS scans (110–1500 *m*/*z*, 15,000 resolution) at 30% normalized collision energy, with a total cycle time of one second. Data analysis was conducted using Thermo Xcalibur Qual Browser and Proteome Discoverer for database matching and TMT quantification. The mass spectrometry proteomics data have been deposited in the ProteomeXchange Consortium via the PRIDE partner repository [28] under dataset identifier PXD048934.

### 2.5. Membrane Proteomics

Three biological replicates of each cell type were performed. Cellular debris was collected, and membrane proteins were isolated using the commercial Membrane Protein Extraction Kit from BestBio Company, China. Protein concentrations were evaluated via the bicinchoninic acid (BCA) assay. For trypsin digestion, 50 micrograms of protein from each sample were treated with ProteaseMAX Surfactant (trypsin enhancer from Promega), following the instructions provided by the manufacturer. The mixture began with 2.0 µL of 1% ProteaseMAX surfactant in 50 mM NH_4_HCO_3_ added to 93.5 µL of the protein sample. This was followed by the addition of 1 µL of 0.5 M DTT, and the mixture was incubated at 56 °C for 20 min. Next, 2.7 µL of 0.55 M of iodoacetamide was added, and the sample was incubated in the dark at room temperature for 15 min. Post-incubation, proteins were precipitated using cold acetone, redissolved in 97.2 µL of 100 mM TEAB buffer, and digested with 1.0 µL of 1% ProteaseMAX surfactant and 1.8 µL of 1 µg/µL trypsin at 37 °C for 3 h. Tryptic peptides were then labeled with TMT reagent, and the labeling reaction was quenched with hydroxylamine. After pooling TMT-labeled peptides from different groups, the mixture was cleaned with an HLB column and fractionated into 10 fractions by high-pH reverse-phase chromatography.

For LC-MS/MS analysis, peptides were eluted over a 120-min gradient at a flow rate of 0.300 µL/min using a Thermo UltiMate 3000 nano-HPLC system connected to a Thermo Orbitrap Fusion Lumos mass spectrometer. The separation was achieved on a homemade fused silica capillary column (75 µm ID, 150 mm length; Upchurch, Oak Harbor, WA, USA) packed with C-18 resin (300 A, 3 µm, Varian, Lexington, MA, USA). Mobile phase A consisted of 0.1% formic acid, and mobile phase B consisted of 80% acetonitrile with 0.1% formic acid. The mass spectrometer operated in data-dependent acquisition mode, overseen by Xcalibur 4.1 software. It commenced with a full-scan mass spectrum in the Orbitrap (350–1800 *m*/*z*, 120,000 resolution), followed by several rounds of data-dependent MS/MS (110–1500 *m*/*z*) and MS3 scans (100–500 *m*/*z*) at 65% normalized collision energy, each within a cycle time of one second. Analysis of each mass spectrum was performed using Thermo Xcalibur Qual Browser and Proteome Discoverer for database searching and TMT quantification. The compiled mass spectrometry proteomics data were submitted to the ProteomeXchange Consortium via the PRIDE partner repository [28] under dataset identifier PXD048934.

### 2.6. Differential Expression Analysis for Proteomics

The protein expression data by global and membrane proteomics were log2 transformed before differential expression analysis was performed using moderated *t*-tests in the R package limma (version 3.54.2) [29]. Differentially expressed proteins (DEPs) were identified based on the cutoffs of FDR-adjusted *p*-values and fold changes (specified in the text and figures) of protein expression between two cell types. Subsequently, functional enrichment analysis was conducted on up- and downregulated proteins by using EnrichR [30] or DAVID [31,32] for gene ontology (GO) and relevant pathways. Regarding the background gene sets, for EnrichR, we used the default reference gene set, which is the complete transcriptome of 20,625 default background genes; for DAVID (used for K-means clustering analysis of the membrane proteomics data), the background set comprised all of the 4327 detected membrane proteins as listed in Supplementary Table S2: Membrane Proteomics. The GO terms and pathways with FDR-adjusted *p*-value less than 0.05 were identified and plotted.

### 2.7. Comparative Analysis of Published Datasets and the Dataset from Our Study

MDSC single-cell RNA sequencing data were retrieved from the Gene Expression Omnibus (GEO) under accession number GSE210963 from Savardekar et al. [33]. Normalized proteomics data of human monocytes and MDSC were downloaded from Ramil et al. [34]. The identification of overlapping proteins between different proteomics datasets was achieved by utilizing the Merge function in R. The bulk RNA sequencing data of monocytes from healthy donors and rectal cancer were obtained from the GEO under the accession number GSE221925 from Larionova et al. [35]. The heatmap was plotted based on the log-transformed counts per million (CPM) expression level of the genes encoding the protein markers. The *p*-values were calculated using the Wilcoxon rank sum test. TCGA RNA-Seq analyses comparing normal versus tumor tissues (including metastatic tumors) for pancreatic adenocarcinoma, skin cutaneous carcinoma, breast invasive carcinoma, and colon adenocarcinoma were conducted using TNMplot [36]. The analysis computes the average expression of the signature for each individual patient to construct in a combined plot. Statistical significance was determined by a two-tailed Mann–Whitney test, with *p* < 0.05 considered significant.

## 3. Results

### 3.1. Confirming the iPMN/iMDSC Model and the Suppression of T Cells by iMDSC

**Figure 1:**
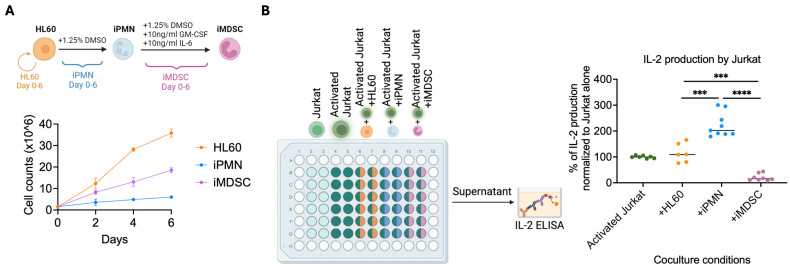
Confirming the iPMN/iMDSC model and the suppression of T cells by iMDSCs. (**A**) Experiment design (upper) and results (lower) for viable cell numbers of HL60, iPMNs, and iMDSCs in a 6-day duration. Viable cells were confirmed by exclusion of Trypan Blue stain. Data represent mean ± SD. (**B**) Experiment design and results for Jurkat–myeloid co-culture assay and ELISA of IL-2 in the medium. *** *p* < 0.001, **** *p* < 0.0001, Mann–Whitney test. Each experiment was repeated at least three times with consistent results.

The cell model used in this study to compare PMNs and MDSCs at the protein expression level was based on our previous report, where we used the human promyelocytic cell line HL60 to derive induced polymorphonuclear neutrophils (iPMNs) and induced MDSCs (iMDSCs) [21]. Briefly, iPMNs were induced by subjecting HL60 cells to 1.25% DMSO over 6 days, while iMDSCs were induced by treating iPMN with DMSO plus 10 ng/mL of GM-CSF and IL-6 for another 6 days. Consistent with our previous report [21], the viable cell numbers followed the trend of HL60 > iMDSC > iPMN (Figure 1A). We further confirmed the immunosuppressive activity of iMDSCs by assessing their impact on IL-2 secretion from activated T cells. HL60, iPMNs, or iMDSCs were co-cultured with anti-CD3/anti-CD28-activated Jurkat cells at a 1:1 ratio for 24 h, followed by IL-2 level measurement in the medium. Consistent with our previous report [21], HL60 did not alter IL-2 secretion from Jurkat, iPMNs stimulated IL-2 production, yet iMDSCs dramatically reduced IL-2 secretion from Jurkat, confirming that iMDSCs were bona fide immunosuppressive myeloid cells (Figure 1B). These results support using this cell line model system to identify MDSC-associated gene expression patterns.

### 3.2. Global Proteomic Profiling of iPMNs and iMDSCs

**Figure 2:**
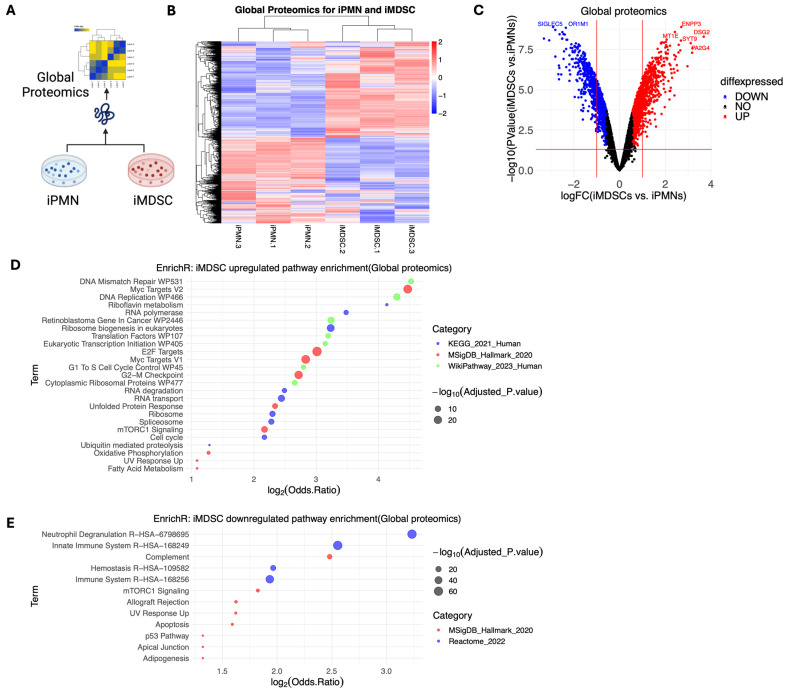
Global proteomic profiling of iPMNs and iMDSCs. (**A**) Schematic of the global proteomics experiment. (**B**) Heatmap of the hierarchical clustering of significantly differentially expressed proteins between iPMNs and iMDSCs (|log2 fold change(iMDSC/iPMN)| > 0.58, *p* < 0.05, FDR < 0.05). (**C**) Volcano plot of all detected proteins in iPMNs and iMDSCs, with red lines representing log2|fold change(iMDSC/iPMN)| = 0.58 and *p* < 0.05. (**D**) Pathways significantly enriched by upregulated proteins in iMDSCs relative to iPMNs, analyzed with EnrichR. (**E**) Pathways significantly enriched by downregulated proteins in iMDSCs relative to iPMNs, analyzed with EnrichR. In (**D**,**E**), adjusted *p*-value cutoff is 0.05; the size, location, and color of the dots represent −log10 (adjusted *p*-value), log2 (odds ratio), and the pathway database, respectively.

iPMNs and iMDSCs were profiled for global proteomics with mass spectrometry (Figure 2A). A total of 4669 proteins were detected, 1953 of which exhibited significant differential expression between the two cell types (absolute fold change ≥ 1.5, false discovery rate (FDR) < 0.05) (Figure 2B, Supplementary Table S1). Among them, 1097 proteins were upregulated and 856 proteins downregulated in iMDSCs. The top upregulated proteins included ENPP3, DSG2, MT1E, SYT9, PA2G4, and EFCAB6, and the top downregulated proteins included SIGLEC5, OR1M1, CRISPLD2, and ITM2B (Figure 2C). Pathway enrichment analysis of proteins upregulated in iMDSCs showed that, compared with iPMNs, iMDSCs were enriched for pathways related to DNA replication, cell cycle, E2F and Myc targets, ribosome biogenesis, protein translation, and anabolic metabolism (Figure 2D). This result was consistent with the higher survival and proliferation of iMDSCs than iPMNs (Figure 1A). Correspondingly, pathway enrichment analysis of proteins downregulated in iMDSCs (upregulated in iPMNs) identified pathways involved in neutrophil degranulation and innate immunity, concordant with the immunosuppressive activity of iMDSCs (Figure 2E). These results provide a comprehensive view of the distinct protein expression pattern of iPMNs and iMDSCs, which correlates with the cellular activities of the two cell types.

### 3.3. Membrane Proteomic Profiling of HL60, iPMN, and iMDSC

**Figure 3:**
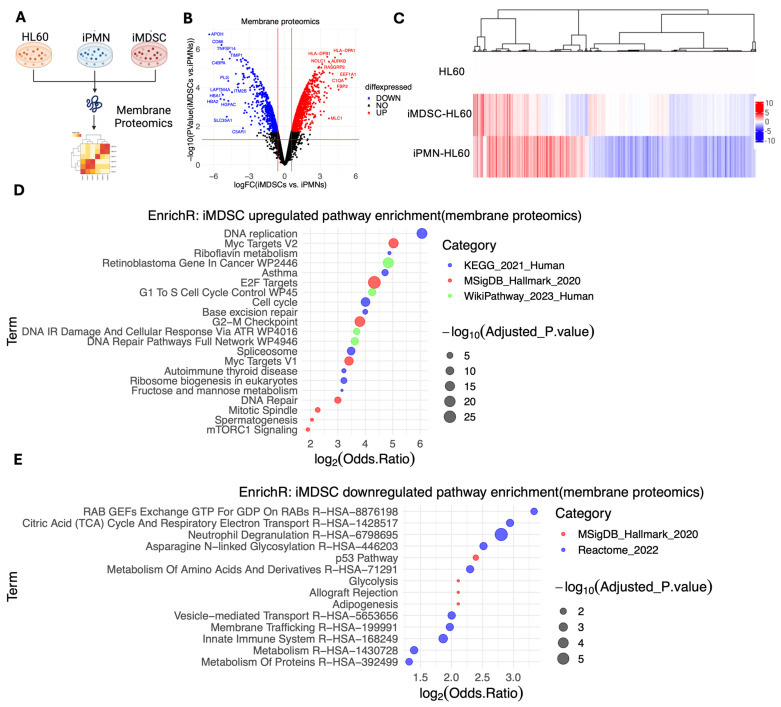
Membrane proteomic profiling of HL60, iPMN, and iMDSC. (**A**) Schematic of the membrane proteomics experiment. (**B**) Volcano plot of all detected proteins in iPMNs and iMDSCs, with red lines representing log2|fold change(iMDSC/iPMN)| = 0.58 and *p* < 0.05. (**C**) Heatmap showing the protein expression in HL60, iMDSCs and iPMNs normalized to the value in HL60. (**D**) Pathways significantly enriched by upregulated proteins in iMDSCs relative to iPMNs, analyzed with EnrichR. (**E**) Pathways significantly enriched by downregulated proteins in iMDSCs relative to iPMNs, analyzed with EnrichR. In (**D**,**E**), adjusted *p*-value cutoff is 0.05; the size, location, and color of the dots represent −log10(adjusted *p*-value), log2 (odds ratio), and the pathway database, respectively.

Proteomic profiling focused on the cell membrane may help enrich protein markers, making them more translatable to clinical applications. We performed a targeted membrane proteomic analysis using mass spectrometry (Figure 3A). This method detected 4298 proteins, 1744 of which displayed marked differential expression between iMDSCs and iPMNs (absolute fold change ≥ 1.5, *p* < 0.05, FDR < 0.05). Among these proteins, 1129 proteins were upregulated and 597 proteins were downregulated in iMDSCs compared with iPMNs (Figure 3B,C, Supplementary Table S2). Pathway enrichment by the upregulated and downregulated proteins showed results consistent with the enrichment from the global proteomics analysis: the upregulated proteins in iMDSCs were enriched for DNA replication, cell cycle, E2F and Myc targets, and mTORC1 signaling (Figure 3D), whereas the downregulated proteins in iMDSC were enriched for neutrophil degranulation, the p53 pathway, and the innate immune system (Figure 3E). This consistent pattern between global proteomics and membrane proteomics confirmed the validity of the membrane proteomics method.

### 3.4. A 41-Protein Signature High in iMDSC but Low in iPMN and HL60

**Figure 4:**
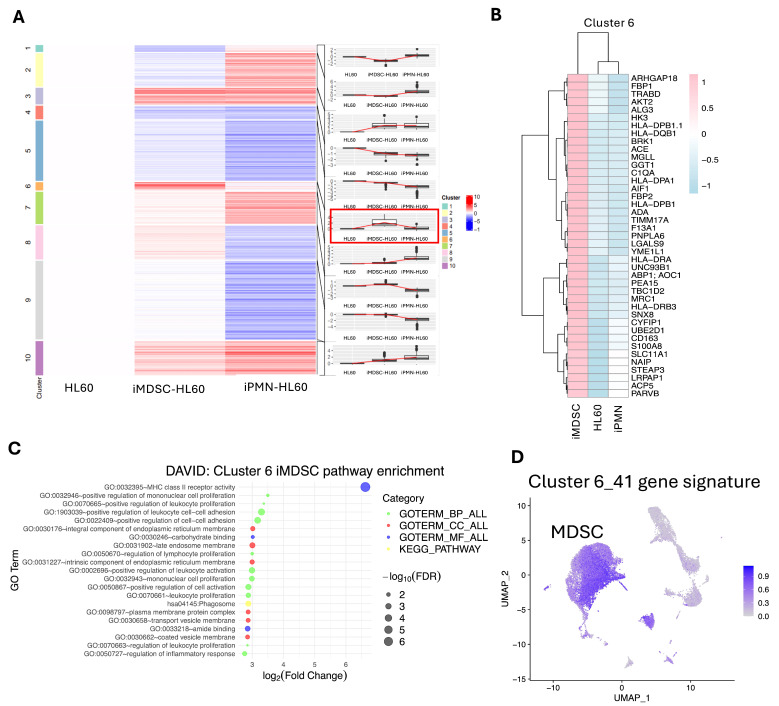
A 41-protein signature high in iMDSCs but low in iPMNs and HL60. (**A**) K-means clustering analysis (k = 10, converged) of the membrane proteomics data highlighting the distinct protein expression patterns of each cluster in HL60, iPMN, and iMDSC. (**B**) Heatmap of log2-transformed expression values of 41 proteins of cluster 6 in iMDSCs, HL60, and iPMNs. (**C**) Top enriched pathways by genes corresponding to cluster 6 proteins using DAVID pathway analysis. Dot size and color reflect the significance and categories. (**D**) UMAP (uniform manifold approximation and projection) plots depicting the average expressions of the log-transformed normalized 41-gene signatures corresponding to the cluster 6 proteins in MDSCs and other cells based on the Savardekar et al. scRNA-Seq dataset.

To resolve distinct expression patterns of the membrane proteins among the three cell statuses, we applied K-means clustering analysis of the membrane proteomics data for proteins which showed a significant difference between any pairwise comparison, iMDSC vs. HL60, iPMN vs. HL60, or iMDSC vs. iPMN (Figure 4A, Supplementary Table S3). Among the 10 clusters, cluster 6, with 41 proteins, drew the most interest because it had the unique pattern of containing proteins markedly upregulated in iMDSCs compared to both HL60 and iPMNs (Figure 4A,B). Interestingly, many proteins in cluster 6 have been reported to function in MDSCs or other immunosuppressive myeloid cells. For instance, S100A8, a member of the calcium-binding S100 protein family, contributes to an autocrine loop facilitating MDSC accumulation [37]. ACP5 presence is noted in bone metastasis-derived MDSCs [38]. CD163 is a well-recognized marker for M2-polarized macrophages, but CD163 is also the marker for MDSCs in Waldenstrom’s macroglobulinemia [39]. FBP1 upregulation has been linked to enhanced immunosuppressive capabilities of MDSCs [34]. AIF1 and MRC1 (also known as CD206), while not specifically tied to MDSCs, are associated with M2-polarized macrophages, which monocytic MDSCs can differentiate into in vivo [40,41]. GGT1 upregulation in MDSCs is required for their immunosuppressive function [42].

In addition to these obvious connections to known MDSC-relevant proteins, pathway enrichment analysis of genes in cluster 6 revealed a significant enrichment of MHC-II receptor activity in iMDSCs (Figure 4C). This enrichment was due to the upregulation in iMDSCs of a number of human HLA class II proteins such as HLA−DPA1, HLA−DPB1, HLA−DPB1.1, HLA−DQB1, HLA−DQB1, HLA−DRA, and HLA−DRB3 (Figure 4B). This striking result is consistent with the previous finding that MHC class II expression on MDSCs was required to induce tolerance of CD4^+^ T cells [43]. To further confirm the upregulated expression of cluster 6 proteins in MDSC-like cells, we examined their expression at the single-cell mRNA level in a human MDSC single-cell RNA-Seq dataset, which profiled CD11b^+^ CD33^+^ HLA-DR^lo/−^ MDSCs isolated from five patients with cancer (three with melanoma, one with head and neck cancer, and one with breast cancer) [33]. The 41-gene signature was expressed at a much higher level in MDSCs than other cells (Figure 4D). Together, this analysis reveals a protein expression signature that shows distinct upregulation in iMDSCs compared with iPMNs or HL60, and many proteins in the signature may contribute to the immunosuppressive function of iMDSCs.

### 3.5. Integration of Global and Membrane Proteomics to Find Shared Proteins

**Figure 5:**
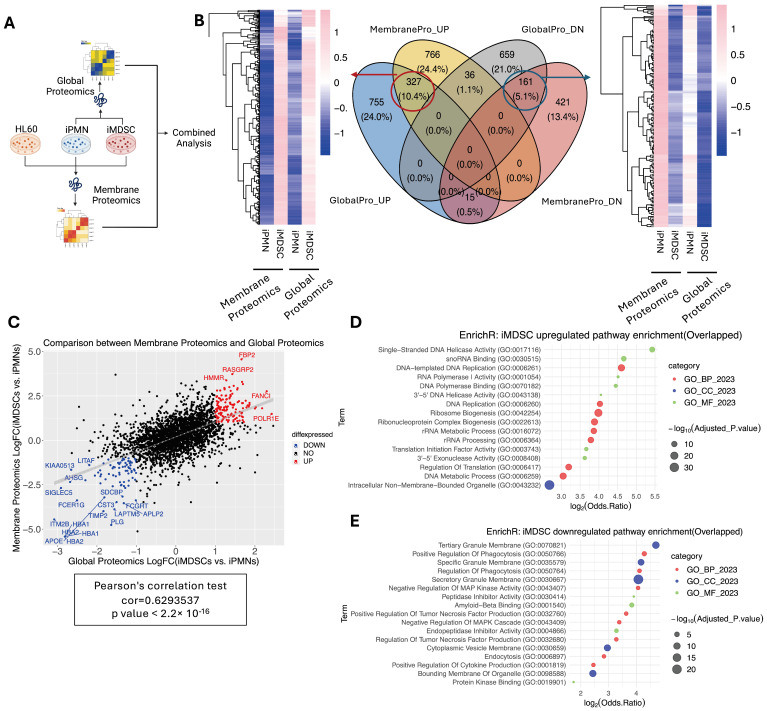
Integration of global and membrane proteomics to find shared proteins. (**A**) Diagram depicting the integrative analysis of global and membrane proteomics. (**B**) Venn diagram pinpointing and heatmaps displaying the consistently upregulated (327) and downregulated (161) proteins in iMDSCs compared with iPMNs (|log2(fold change)| > 0.58). (**C**) Strong correlation between global and membrane proteomics in terms of FCs of proteins in iMDSCs compared to iPMNs, where red dots label significantly co-upregulated proteins, while blue labels indicate co-downregulated proteins in iMDSCs. (**D**) Top enriched GO terms for co-upregulated proteins (adjusted *p* < 0.05), using EnrichR. (**E**) Top enriched GO terms for co-downregulated proteins in iMDSCs (adjusted *p* < 0.05), using EnrichR.

To identify proteins upregulated or downregulated in iMDSCs in both proteomics datasets, we focused on the difference between iMDSCs and iPMNs because these two cell types were profiled in both experiments (Figure 5A). A four-way Venn diagram of upregulated (log2(fold change) > 0.58) and downregulated (log2(fold change) < −0.58) proteins narrowed the dataset down to 327 proteins consistently upregulated and 161 proteins consistently downregulated in iMDSCs compared with iPMNs in both datasets (Figure 5B). Moreover, the iMDSC/iPMN fold changes for the 3108 proteins detected in both datasets correlated very well (Figure 5C), indicating general agreement between the two detection methods. Pathway analysis confirmed that the consistently upregulated proteins in iMDSCs were enriched for pathways and functions involved in cell proliferation and active metabolism, such as DNA replication, ribosome biogenesis, and translation initiation factor activity (Figure 5D), whereas consistently downregulated proteins were enriched for many functions related to phagocytosis and classical neutrophil activities, such as positive regulation of phagocytosis, granule membranes (specific, secretory, and tertiary), cytokine production, and tumor necrosis factor production (Figure 5E). The results are concordant with the proliferative and immunosuppressive behaviors of iMSDCs, which are feeble in iPMNs.

### 3.6. Consistently Upregulated MDSC-Associated Proteins across Datasets

**Figure 6:**
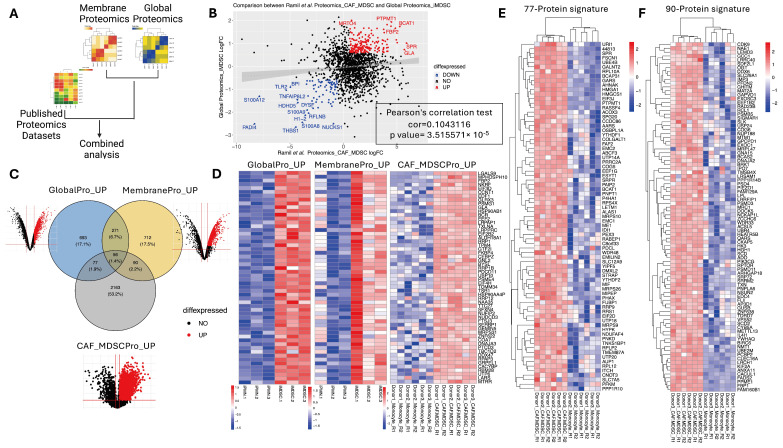
Consistently upregulated MDSC-associated proteins across datasets. (**A**) Diagram depicting the comparative analysis of global and membrane proteomics in our study and the published Ramil et al. proteomics dataset (CAF_MDSC). (**B**) A scatter plot of the fold changes of the proteins detected in both our global proteomics and the proteomics of Ramil et al. Significantly co-upregulated and co-downregulated proteins are labeled in red and blue, respectively. (**C**) A Venn diagram showing the intersections of upregulated proteins identified across global proteomics, membrane proteomics, and Ramil dataset. (**D**) Heatmap of the 56 proteins consistently upregulated across global proteomics, membrane proteomics, and Ramil dataset. (**E**) Heatmap of the 77 proteins upregulated in both global proteomics and Ramil dataset. (**F**) Heatmap of the 90 proteins upregulated in both membrane proteomics and Ramil dataset.

To cross-validate the MDSC-associated proteins emerging from our study with other studies, we identified one recent publication by Ramil et al. that profiled the proteome of human monocytes and monocyte-derived MDSCs induced with GM-CSF and co-culture of cancer-associated fibroblasts [34] (Figure 6A). The fold changes of proteins detected in our global proteomics data (iMDSC/iPMN) and Ramil’s data (CAF-MDSCs/monocytes) showed a statistically significant correlation (Figure 6B); nonetheless, the correlation coefficient was moderate, presumably due to the differences in the exact cell types profiled in the two studies. Next, we focused on finding consistently upregulated proteins in MDSCs across the two studies. Based on the Venn diagram, 56 proteins were upregulated in all three datasets (Figure 6C,D, Supplementary Table S4), 77 proteins were upregulated in our global proteomics and Ramil’s datasets (Figure 6E, Supplementary Table S5), and 90 proteins were upregulated in our membrane proteomics and Ramil’s dataset (Figure 6F, Supplementary Table S6). These in silico cross-validated proteins are likely to have higher chances of being experimentally validated as MDSC markers in follow-up studies.

### 3.7. A 38-Protein MDSC Signature Based on Overlap Analysis, Functional Assessment, and Clinical Association

**Figure 7:**
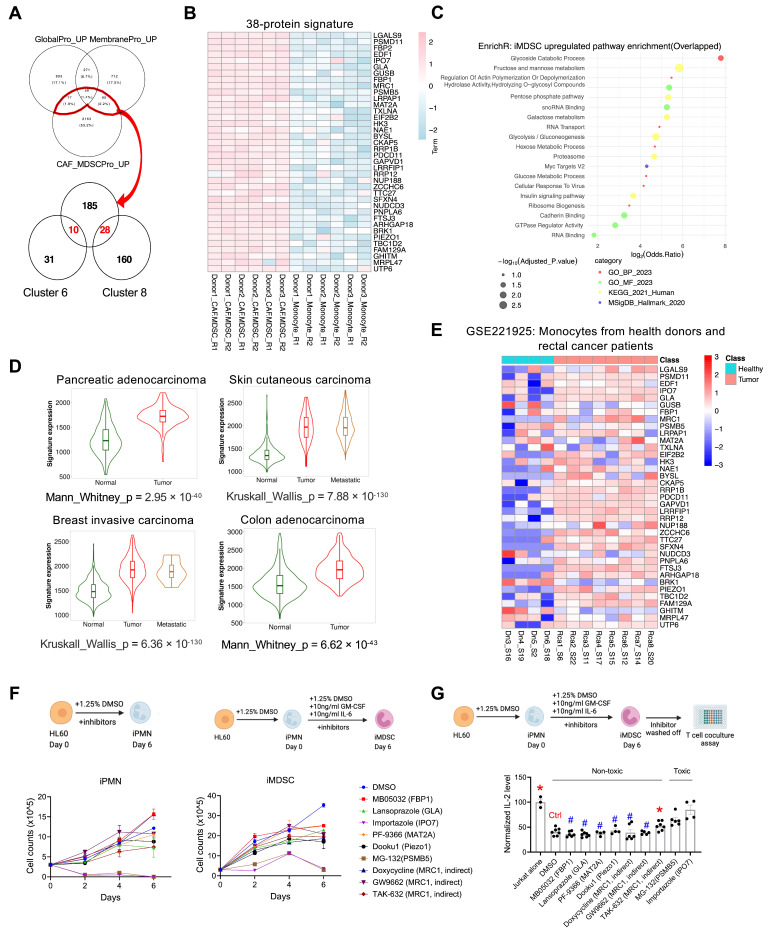
A 38-protein MDSC signature based on overlap analysis and clinical association. (**A**) Venn diagrams to narrow down upregulated proteins associated with MDSCs. (**B**) Heatmap of the 38 proteins between normal monocytes from healthy donors and CAF-induced MDSCs based on Ramil dataset. (**C**) Top enriched pathways based on the 38-protein signature (adjusted *p* < 0.05) using EnrichR. (**D**) Normalized expression level of the 38-gene MDSC signature in normal tissue, primary tumors, and metastases (when available) for four cancer types, based on TCGA data, plotted using TNMplot. (**E**) Heatmap of average log-transformed normalized expressions of 37 gene signatures (FBP2 is absent in the dataset) between normal monocytes from healthy donors and their pathogenic counterparts from rectal cancer patients derived from the bulk RNA-Seq dataset (GSE221925). (**F**) Experimental design (**top**) and corresponding results (**below**) assessing the cell number changes of iPMNs and iMDSCs in the presence of DMSO (vehicle control) or various inhibitors. (**G**) Experimental design (**top**) and results (**below**) assessing the effect of the inhibitors on iMDSC activity to attenuate IL-2 production from stimulated Jurkat cells. IL-2 levels in the medium were measured with ELISA. In (**F**,**G**), data represent mean ± s.e.m. * *p* < 0.05, # *p* > 0.05, two-sided *t*-test. Each experiment was conducted at least three times, yielding consistent results.

We further narrowed down the list of proteins upregulated in MDSCs based on overlapping the proteins upregulated in both our proteomics data and Ramil’s data (227 proteins, 56 + 77 + 90) with the ones in cluster 6 and cluster 8 from the K-means analysis of the membrane proteomics (because proteins in clusters 6 and 8 have the highest levels in iMDSCs compared with HL60 or iPMNs). A total of 38 proteins emerged (Figure 7A,B, Supplementary Table S7).

The pathway enrichment analysis of these proteins indicated a pronounced activation of various metabolic pathways, including glycolysis/gluconeogenesis, fructose and mannose metabolism, the pentose phosphate pathway, and the glycoside catabolic process (Figure 7C), consistent with the report that glycolysis facilitates the expansion of MDSCs in tumor-bearing hosts by inhibiting ROS-mediated apoptosis [44]. This 38-protein signature was analyzed at the mRNA level with the transcriptomics data from The Cancer Genome Atlas (TCGA) using TNMplot [36]. We selected a few cancer types known for high MDSC infiltration: pancreatic adenocarcinoma, skin cutaneous carcinoma, breast invasive carcinoma, and colon adenocarcinoma [45,46,47,48]. This signature was markedly upregulated in tumor tissues compared to normal tissues (Figure 7D), underscoring its relevance in the tumor microenvironment. To assess the expression pattern of these genes in isolated cells instead of tissues, we used RNA-Seq data from a previous study that profiled normal monocytes from healthy donors and tumor-associated monocytes from patients with rectal cancer [35]. The transcripts that encode the 37 proteins (FBP2 is absent) were also generally expressed at higher levels in tumor-associated monocytes (many were likely monocytic MDSCs) than in normal monocytes (Figure 7E, Supplementary Table S8).

To identify putative proteins from the 38-protein signature that may functionally contribute to MDSCs, we selected seven proteins based on their previously reported connections with tumor immune regulation (especially immunosuppression) and the availability of relevant pharmacological inhibitors (Supplementary Table S9). As mentioned above, FBP1 upregulation has been linked to enhanced immunosuppressive capabilities of MDSCs [34]. Upregulation of alpha-galactosidase A (GLA) is associated with poor prognosis and immune infiltration in glioma [49]. Importin 7 (IPO7), part of the karyopherin-β protein family, influences tumorigenesis and progression by facilitating the nuclear import of oncoproteins. Studies in cervical and pancreatic cancers indicate that elevated IPO7 expression correlates with poor prognosis and decreased CD8 T cell infiltration in cervical cancer and adverse outcomes in pancreatic cancer patients [50,51]. Elevated methionine adenosyltransferase II alpha (MAT2A) levels in monocytes/macrophages from various sources correlate with increased methionine cycle activity and maintenance of TAMs’ immunosuppressive phenotype via the RIP1-H3K4 methylation axis [52]. PIEZO1 is a mechanosensitive ion channel protein that promotes cancer progression and MDSC expansion [53]. PSMB5, part of the proteasome β subunits (PSMB) family and crucial in the ubiquitin-proteasome system, is linked with oncogenic traits and immunosuppression in M2 macrophages; high PSMB5 levels predict poor survival in breast cancer [54]. Lastly, mannose receptor C-type 1 (MRC1, also known as CD206), a marker for M2 macrophages, promotes tumor-supportive functions. Analysis of MC38 tumors in mice treated with anti-PD-1 therapy revealed high MRC1 expression in non-responders [55].

We inhibited these seven proteins with nine inhibitors, including six direct inhibitors for FBP1, GLA, IPO7, MAT2A, PIEZO1, and PSMB5 and three indirect inhibitors for MRC1 (Supplementary Table S9). We examined whether the inhibitors affected the proliferation of iPMNs and iMDSCs and whether they affected how much iMSDCs attenuated IL-2 production from stimulated Jurkat cells. The inhibitors were added simultaneously as the inducing factors for iPMNs and iMDSCs. MG-132 (proteasome inhibitor for PSMB5) and Importazole (IPO7 inhibitor) were toxic to both iPMNs and iMDSCs, whereas other inhibitors were generally tolerated by iPMNs and iMSDCs **(**Figure 7F). To assess the inhibitors’ effect on immunosuppression, we washed off the inhibitors from iMDSCs before co-culturing iMDSCs and stimulated Jurkat cells at a 1:1 ratio for 24 h, followed by IL-2 measurement with ELISA. This assay showed that TAK-632 (RAF inhibitor, indirectly inhibiting MRC1) was the only non-toxic agent and showed partially restored IL-2 levels (Figure 7G), suggesting that TAK-632 may mitigate the suppressive activity of iMDSCs on T cells.

## 4. Discussion

MDSCs are a heterogeneous group of cells that can suppress T-cell mediated antitumor immunity and promote tumor growth [6]. The current study identified several protein signatures differentially expressed between MDSCs and PMNs based on the HL60 cell line model, culminating with the 38-protein signature that shows higher expression levels in tumors compared with normal tissues. More importantly, these proteins and their corresponding transcripts are expressed at higher levels in tumor-infiltrating MDSC-like cells compared with healthy monocytes (Figure 7B,E), suggesting the disease-associated specificity of the signature. Future research should focus on elucidating how the genes within the signature directly contribute to the phenotypic or functional characteristics of MDSCs. Such studies could involve targeted gene manipulation to observe changes in MDSC behavior and impacts on tumor growth and immune evasion. The protein signatures identified in our study provide a hypothesis-generating platform to identify novel biomarkers and therapeutic targets for MDSCs.

Given the role of MDSCs in cancer, strategies aimed at inhibiting their function or reducing their accumulation could effectively enhance antitumor immunity. The proteins identified in this study could be potential targets for such strategies. Exploring inhibitors that specifically target these proteins or their pathways might disrupt the immunosuppressive activity of MDSCs, thereby enhancing the efficacy of existing cancer therapies, including immunotherapies. In this study, we only focused on seven proteins from the 38-protein signature and used inhibitors that directly or indirectly dampen these proteins to conduct an initial screening for the effect on T cell suppression by iMDSCs. While the result suggests that TAK-632 may reduce the suppressive activity of iMDSCs on T cells, we should note that TAK-632 was only reported to indirectly lower MRC1 expression by inhibiting macrophage polarization toward the M2 phenotype [56]. Future studies are warranted to elucidate the mechanism of how TAK-632 alters MDSC functions and, more importantly, identify and validate other biomarkers and therapeutic targets from the signature.

The role of MDSCs extends beyond cancer, as they are implicated in various other pathological conditions, including autoimmune diseases and chronic infections [57,58]. Future research could also explore the application of the 38-protein signature in these contexts, investigating whether these proteins have similar roles in MDSC-mediated immune regulation in other diseases. The moderate correlation coefficient in the correlation analysis of our data and the data of Ramil et al. may stem from the heterogeneous nature of HL60-induced MDSCs, which consist of both CD14+ monocytic (Mo-MDSCs) and CD15+ polymorphonuclear (PMN-MDSCs) cells [21], whereas the MDSCs in Ramil et al. should be mainly Mo-MDSCs [34]. Therefore, a precise understanding of how these proteins are differentially regulated in PMN-MDSCs and Mo-MDSCs could lead to more targeted therapeutic approaches. A technical limitation of our study is that we did not have a designated step to remove dead cells other than removing supernatant and washing the cell pellets with PBS before extracting the proteins for proteomic profiling. sFuture experiments should include a step of dead cell removal before profiling.

The implications of this study are broad, offering new perspectives on tumor immunology and potential therapeutic approaches. As research continues to unravel the complexities of MDSCs, the insights gained from studies like this will be pivotal in guiding future therapeutic interventions and improving patient outcomes in cancer and other MDSC-related diseases.

## Data Availability

The mass spectrometry proteomics data have been deposited to the ProteomeXchange Consortium via the PRIDE partner repository with the dataset identifier PXD048934. All other data that support the findings of this study are available from the corresponding authors upon reasonable request.

## Supplementary Materials

The following supporting information can be downloaded at: https://www.mdpi.com/article/10.3390/cells13100795/s1, Table S1: Global Proteomics; Table S2: Membrane Proteomics; Table S3: Membrane Proteomics_10 clusters; Table S4: 56-Protein signature; Table S5: 77-Protein signature; Table S6: 90-Protein signature; Table S7: 38-Protein signature; Table S8: 38-Protein signature in Larionova et al; Table S9: iMDSC Inhibitor screening_Drug information.
